# Smek1 deficiency exacerbates experimental autoimmune encephalomyelitis by activating proinflammatory microglia and suppressing the IDO1-AhR pathway

**DOI:** 10.1186/s12974-021-02193-0

**Published:** 2021-06-28

**Authors:** Ruo-Nan Duan, Chun-Lin Yang, Tong Du, Ai Liu, An-Ran Wang, Wen-Jie Sun, Xi Li, Jiang-Xia Li, Chuan-Zhu Yan, Qi-Ji Liu

**Affiliations:** 1 Key Laboratory for Experimental Teratology of the Ministry of Education and Department of Medical Genetics, Cheeloo College of Medicine, School of Basic Medical Sciences, Shandong University, No.44 West Wenhua Road, Jinan, Shandong 250012 People’s Republic of China; 2 Department of Neurology, Qilu Hospital, Cheeloo College of Medicine, Shandong University, Jinan, People’s Republic of China; 3 Department of Neurology, Shandong Provincial Qianfoshan Hospital, Cheeloo College of Medicine, Shandong University, Jinan, People’s Republic of China

**Keywords:** Suppressor of MEK1, Experimental autoimmune encephalomyelitis, Indoleamine 2,3-dioxygenase, Microglia, Interleukin 1β

## Abstract

**Background:**

Experimental autoimmune encephalomyelitis (EAE) is an animal disease model of multiple sclerosis (MS) that involves the immune system and central nervous system (CNS). However, it is unclear how genetic predispositions promote neuroinflammation in MS and EAE. Here, we investigated how partial loss-of-function of suppressor of MEK1 (SMEK1), a regulatory subunit of protein phosphatase 4, facilitates the onset of MS and EAE.

**Methods:**

C57BL/6 mice were immunized with myelin oligodendrocyte glycoprotein 35-55 (MOG_35-55_) to establish the EAE model. Clinical signs were recorded and pathogenesis was investigated after immunization. CNS tissues were analyzed by immunostaining, quantitative polymerase chain reaction (qPCR), western blot analysis, and enzyme-linked immunosorbent assay (ELISA). Single-cell analysis was carried out in the cortices and hippocampus. Splenic and lymph node cells were evaluated with flow cytometry, qPCR, and western blot analysis.

**Results:**

Here, we showed that partial Smek1 deficiency caused more severe symptoms in the EAE model than in controls by activating myeloid cells and that Smek1 was required for maintaining immunosuppressive function by modulating the indoleamine 2,3-dioxygenase (IDO1)-aryl hydrocarbon receptor (AhR) pathway. Single-cell sequencing and an in vitro study showed that Smek1-deficient microglia and macrophages were preactivated at steady state. After MOG_35-55_ immunization, microglia and macrophages underwent hyperactivation and produced increased IL-1β in Smek1^-/+^ mice at the peak stage. Moreover, dysfunction of the IDO1-AhR pathway resulted from the reduction of interferon γ (IFN-γ), enhanced antigen presentation ability, and inhibition of anti-inflammatory processes in Smek1^-/+^ EAE mice.

**Conclusions:**

The present study suggests a protective role of Smek1 in autoimmune demyelination pathogenesis via immune suppression and inflammation regulation in both the immune system and the central nervous system. Our findings provide an instructive basis for the roles of Smek1 in EAE and broaden the understanding of the genetic factors involved in the pathogenesis of autoimmune demyelination.

**Supplementary Information:**

The online version contains supplementary material available at 10.1186/s12974-021-02193-0.

## Introduction

Protein phosphatase 4 (PP4), a highly conserved serine/threonine phosphatase, is a protein complex composed of catalytic subunits and regulatory subunits. Suppressor of MEK1 (SMEK1), a regulatory subunit of the PP4 enzyme, regulates the activity of the PP4 catalytic subunits, leading to dephosphorylation of its target substrates via an unknown mechanism. PP4 is involved in many cellular processes in organisms and regulates several cellular signaling pathways, including nuclear factor kappa B (NF-κB), c-Jun N-terminal kinase, apoptotic signaling, insulin receptor substrate protein 4, and target of rapamycin [1]. Recent findings suggest that PP4 is an essential modulator of T cell proliferation and immune responses by dephosphorylating adenosine monophosphate activated protein kinase (AMPK) [2]. Furthermore, rectal prolapse and colitis (similar to Crohn’s disease) spontaneously develop in CD4-Cre PP4^fl/fl^ mice owing to the impaired function of T regulatory cells [3]. As a subunit of PP4, Smek1 has been found to play roles in chemotaxis [4], neurogenesis [5, 6], neuronal differentiation [7], and aging [8]. However, the effects of SMEK1 on the immune system and autoimmune diseases remain unclear.

Experimental autoimmune encephalomyelitis (EAE), a disease model of multiple sclerosis (MS), is characterized by demyelination resulting from aberrant immune functions in both the immune system and central nervous system (CNS). Microglia, together with macrophages and astrocytes, are the most abundant immune cells in active MS lesions of the brain and spinal cord [9]. Furthermore, microglia are activated in MS normal-appearing white and gray matter [10–12]. A transcriptome analysis has confirmed the pivotal role of microglia in MS by showing that 48 out of 81 MS-associated risk allele genes are preferentially expressed in microglia [13]. In an EAE model, microglia are regarded as the antigen-presenting cells [14] and secrete both cytokines and chemokines to aggravate MS and EAE [15]. Most studies have focused on biological processes in proinflammatory microglia after MS/EAE onset; however, few studies have found pre-existing microglial abnormalities prior to disease development that may cause predisposition to the disease.

Interferon γ (IFN-γ) also plays a pleiotropic role in EAE and MS. Administration of both IFN-γ and anti-IFN-γ antibody can exacerbate clinical symptoms [16–22]. Conventionally, IFN-γ aggravates neuroinflammation through many aspects. It is proved that IFN-γ induces blood-brain barrier (BBB) leakage in an experimental viral encephalitis mouse model [23]. IFN-γ facilitates the activation of microglia and astrocytes and stimulates the expression of major histocompatibility complex (MHC) [24, 25]. Recently, a study focusing on the chronic neuroinflammation phase has suggested a protective role for IFN-γ [26]. In a study of EAE, symptoms have been shown to be ameliorated in mice injected with IFN-γ–modified dendritic cells (DCs), and the DC–IFN-γ pathway is correlated with EAE dependent on indoleamine 2,3-dioxygenase (IDO1) [27]. IDO is able to catalyze the degradation of tryptophan into kynurenine, which, in turn, gives rise to impairment of CD8+ T cells activation and so enhance development of some diseases [28]. Since accumulating evidence has indicated the beneficial effects of IFN-γ in EAE, low IFN-γ may be a risk factor for disease development.

In this study, we discovered that a reduction in Smek1 function caused preactivation of myeloid cells and severe symptoms in an EAE model. We also identified a novel cluster of proinflammatory colony-stimulating factor 1-positive (Csf1^+^) microglia that highly express interleukin 1β (IL-1β) and chemokines in Smek1^-/-^ mice. This finding strongly suggests that Smek1 deficiency may increase CNS vulnerability to neuroinflammatory diseases. Further analysis showed that T helper (Th) 1 cells in Smek1^-/+^ mice secreted lower IFN-γ than those in control mice, leading to suppression of the IFN-γ-dependent IDO1-aryl hydrocarbon receptor (AhR) pathway and promotion of immune responses in systemic immune organs and the CNS.

## Materials and methods

### Microarray dataset

Public peripheral blood mononuclear immune cell data from MS patients and the unaffected control microarray gene-profiling dataset GSE21942 were downloaded from Gene Expression Omnibus (GEO) using the Illumina Human HT-12 V3.0 platform. Microarray gene profiling datasets of brain tissue from MS brain lesions and control tissue (GSE38010, GSE5839) were downloaded from the Gene Expression Omnibus (GEO) using the Illumina Human HT-12 V3.0 platform.

### Animals

Smek1-floxed mice were generated by delivering a linearized vector to ES cells (C57BL/6) by Cyagen Biosciences. Smek1^fl/+^ Sox2-Cre and Smek1^fl/fl^ Sox2-Cre mice are referred to as Smek1^-/+^ and Smek1^-/-^ mice, respectively. Sox2-Cre mice were from Shanghai Model Organisms Center. Mice were group housed under a 12 h light/dark cycle (light between 07:00 and 19:00) in a temperature-controlled room. All mice were bred and housed under specific pathogen-free conditions at Shandong University.

### Genotyping

Mouse genotyping was performed using mouse tails with the following primers:

F1 (5′-ATTGCTTCCCAGTCTTCACTCTTC-3′),

R1 (5′-TCCCAAAACCACCACAGAACTAAC-3′), and

R3 (5′-GAGATGGCTCAGTGGAGTATTGG-3′). Agarose gel electrophoresis was used to confirm the proliferation products. In wild-type mice and Smek1^-/+^ or Smek1^-/-^ mice, the sequence between F1 and R1 was 296 bp (wild-type allele), and that between F1 and R3 was 427 bp (knockout allele).

### Induction of the EAE model

To induce EAE, female mice at 6–8 weeks old (n = 14 and 17 wild-type and Smek1^-/+^ mice, respectively) were immunized along their back with MOG_35–55_ (2 mg/ml) and *Mycobacterium tuberculosis* (8 mg/ml) dissolved in complete Freund’s adjuvant. Immediately after immunization and 2 days after immunization, mice received an intraperitoneal injection of pertussis toxin (200 ng; List Biological, Campbell, CA) in 100 μL phosphate-buffered saline (PBS). Animals were weighed and evaluated in a blinded fashion for clinical signs. The clinical scores of EAE were assessed every day according to the following criteria: 0, asymptomatic; 0.5, loss of tail tone confined to the tail tip; 1, complete loss of tail tone; 1.5, loss of tail tone and abnormal gait; 2, complete paraparesis in a single hind limb or incomplete paraparesis in bilateral hind limbs; 2.5, complete paraparesis in a single hind limb and incomplete paraparesis in bilateral hind limbs; 3, complete paraparesis in bilateral hind limbs; 4, complete paraparesis in bilateral hind limbs and abnormal fore limb(s); and 5, deceased.

### Histopathology

EAE mice were sacrificed through cardiac perfusion at day 18 postinoculation. Lumbar enlargements of the spinal cord and brain were dissected and fixed in 4% paraformaldehyde for paraffin sectioning. Histopathology assays were performed as previously described [29]. Hematoxylin-eosin (H&E) staining was performed with eosin (F527FA0002, Sangon Biotech) and hematoxylin (F108FA0006, Sangon Biotech). The following antibodies were used: anti-IL-1β (Abcam, ab9722), anti-IBA1 (ServiceBio, GB12105), anti-GFAP (ServiceBio, GB11096), anti-CD4 (ServiceBio, GB13064-1), anti-MMP9 (Proteintech, 10375-2-AP), and anti-CSF1 (Affinity, DF12536). Immunostaining analyses were quantified with Image J.

### qPCR

Total RNA extraction and real-time PCR assays were performed as previously described [30]. The expression of GAPDH was used as an internal control. The primers were designed according to sequences on Ensembl GRCm38.p6. The primers included the following:

IL-1β (homo) 5′-ATGATGGCTTATTACAGTGGCAA-3′ (forward),

5′-CCTCTCTCTAATCAGCCCTCTG-3′ (reverse);

GAPDH (homo) 5′-CCAGGTGGTCTCCTCTGACTT-3′ (forward),

5′- GTTGCTGTAGCCAAATTCGTTGT3′ (reverse);

IL-1β (mus) 5′-GCAACTGTTCCTGAACTCAACT-3′ (forward),

5′-ATCTTTTGGGGTCCGTCAACT-3′ (reverse);

T-bet (mus) 5′-AGCAAGGACGGCGAATGTT-3′ (forward),

5′-GGGTGGACATATAAGCGGTTC-3′ (reverse);

IFN-γ (mus) 5′-ATGAACGCTACACACTGCATC-3′ (forward),

5′-CCATCCTTTTGCCAGTTCCTC-3′ (reverse);

IL-10 (mus) 5′-GCTCTTACTGACTGGCATGAG-3′ (forward),

5′-CGCAGCTCTAGGAGCATGTG-3′ (reverse);

IDO1 (mus) 5′-GCCTCCTATTCTGTCTTATGCAG-3′ (forward),

5′-ATACAGTGGGGATTGCTTTGATT-3′ (reverse);

GAPDH (mus) 5′-AGGTCGGTGTGAACGGATTTG-3′ (forward),

5′-TGTAGACCATGTAGTTGAGGTCA-3′ (reverse).

### Flow cytometry and intracellular cytokine staining

Spleens or lymph nodes were homogenized by crushing with a glass rod in a Petri dish, followed by straining through a 40-μm nylon mesh. Red blood cells of splenic single cell suspensions were lysed with RBC lysis buffer (Biolegend) for 5 min on ice. Isolated spleen and lymph node cell suspensions were stained according to the standard protocols with antibodies against the following molecules: CD4 (Biolegend, 100510), CD3 (eBioscience, 17-0031-81), IFN-γ (Biolegend, 505808), IL-10 (Biolegend, 505009), IL-4 (eBioscience, 12-7041-81), CD11c (eBioscience, 1931037), CD80 (eBioscience, 12-0801-81), CD86 (Biolegend, 105025), MHC-II (eBioscience, 17-5321-81), F4/80 (Biolegend, 123107), and IL-1β (eBioscience, 12-7114-80). For intracellular cytokine staining, cells were stimulated for 5 h at 37 °C with a cell stimulation cocktail (eBioscience). Stimulated cells were then preincubated with PBS containing 0.5% bovine serum albumin and were stained for 30 min at 4 °C with combinations of fluorescently tagged monoclonal antibodies (mAbs). After washing, cells were fixed in 2% paraformaldehyde, permeabilized by using an intracellular fixation and permeabilization buffer set (eBioscience, 88-8824-00) and then stained with mAbs specific for various cytokines. FACS involved the use of the BD FACS Aria II (BD Biosciences). Data were imported into FlowJo (V10.4.1) for further analysis.

### Sample processing for single-cell RNA sequencing

Cerebral cortices (both halves) and hippocampi from 2-month-old mice (N = 2 for each group) were dissected and rinsed in DPBS. Tissues were then processed by using the Adult Brain Dissociation Kit (Miltenyi Biotec). All procedures were performed at room temperature. Centrifugation was performed at 4 °C unless otherwise stated. Gentle MACS Dissociators were used during tissue processing. Library preparation was performed according to the instructions of the 10X Chromium single-cell kit. The libraries were then pooled and sequenced across six lanes on a HiSeq4000.

### Single-cell RNA sequencing

Quality control and data processing were carried out by LC-Bio Technology (Hangzhou, China). 10X genomics raw data were processed by using the Cell Ranger Single-Cell Software Suite (release 2.0), including using cellranger mkfastq for demultiplexing raw base call files into FASTQ files and then using cellranger count for alignment, filtering, barcode counting, and UMI (unique molecular identifier) counting. The reads were aligned to the hg19 reference genome by using a prebuilt annotation package downloaded from the 10X Genomics website. The outputs from different lanes were finally aggregated by using cellranger aggr with the default parameter settings.

We mapped UMIs to genes, followed by removing low-quality cells. Cells were flagged as poor-quality if 1) the number of expressed genes was < 500 and 2) 10% or more UMIs mapped to mitochondrial or ribosomal genes. Cells meeting the latter threshold were usually nonviable or apoptotic. As a result, 5642 and 5041 cells were obtained from the Smek1^-/-^ and wild-type groups, respectively. The data were then normalized. Genes with high variation were identified using FindVariableFeatures. Data scaling was performed prior to dimensional reduction techniques by PCA. PCA dimensionality was determined with the help of the Elbow function. We then used the FindNeighbors and FindClusters functions in the Seurat package for cell clustering analysis and displayed the 2D map using tSNE. RNA-seq datasets can be accessed on GEO: GSE171986.

### Annotating cell clusters

The FindAllMarkers function in Seurat was used to identify unique cluster-specific marker genes. We first identified brain cell clusters with the dominant expression pattern of brain cell markers from the online database and published literature. Putative clusters with common canonical markers were integrated into one cluster. For example, clusters 0 to 3 exhibited different expression patterns but shared common highly expressed markers for microglia. Violin plots, heatmaps, and cluster annotations were carried out in Seurat with the corresponding footnotes.

### Single-cell trajectory analysis and Kyoto Encyclopedia of Genes and Genomes (KEGG) analysis

Single-cell trajectories were constructed by using the Monocle package based on the analysis of the UMI read data. The default settings were used for all other parameters. Pseudotime analysis data were classified into microglial clusters and cell states in different groups. KEGG data were analyzed using DAVID (https://david.ncifcrf.gov/).

### Cell culture, lentivirus, and cell transfection

Human full-length SMEK1 plasmids were selected and inserted into the pLVX-IRES-Puro vector for stable overexpression. SMEK1 RNAi was designed to target the GATTTGTTTGCACAACTAA and ACTTGTATTGGAATTGTTA sequences in the GV248 vector. HMO6 (human microglial cells) were cultured in Dulbecco’s modified Eagle medium with 10% fetal bovine serum. Cells were transfected with short hairpin SMEK1 (shSMEK1) and pLVX-SMEK1 RNA. CON077 and pVLX-IRES-Puro were transfected as negative controls. Cells were screened with puromycin at the appropriate concentration.

### Preparation of splenic mononuclear cells

Female mice were sacrificed at 6–8 weeks of age. The spleen was dissected and ground through a 40-mm nylon mesh. After erythrocytes were osmotically lysed, cells were washed 3 times with PBS and resuspended in 1640 medium. Lipopolysaccharide (LPS; Sangon Biotech, 1 μg/ml) and the anti-CD3 (Biolegend; 1 μg/ml), anti-CD28 (Biolegend; 1 μg/ml), and anti-IFN-γ (Biolegend; 1 μg/ml) antibodies were added to 1640 medium. Cells were then cultured for 48 h.

### Western blot analysis

Cells were lysed by radioimmunoprecipitation assay lysis buffer (Bioteke) containing 1% protease inhibitor and 1% phosphatase inhibitors. The protein concentrations were determined by the bicinchoninic acid method (Thermo Scientific). The details of the immunoblotting assay were previously described. The following antibodies were used: anti-SMEK1 (Sigma, HPA002568), anti-phospho-STAT1 (Y701) (Cell Signaling Technology, #7649), anti-phospho-p65 (Cell Signaling Technology, #3039), anti-p65 (Cell Signaling Technology, #8242), anti-GAPDH (Proteintech, 60004-1-Ig), anti-STAT1 (Abways, AY4260), anti-AhR (Abways, CY3431), and anti-IDO1 (Santa Cruz, sc-365086). The lanes were analyzed by ImageJ.

### Blood–brain barrier assay

A blood–brain barrier assay was carried out at the EAE peak stage. Sodium fluorescein (Sigma) was dissolved in sterile normal saline at 10 mg/ml. Sodium fluorescein (100 μl) was administered by intraperitoneal injection (n = 3 in each group). Control mice were injected with PBS. Mice were sacrificed 30 min after administration. Blood serum was collected from eyeballs. Brains were dissected, weighed, and homogenized in PBS. The homogenate was centrifuged at 10000 g for 5 min at 4 °C. Brain supernatant (100 μl) and serum (50 μl) were measured at an excitation wavelength of 493 nm and an emission wavelength of 519 nm.

### Statistical analysis

Data from mice and cell lines are presented as the mean ± SEM and mean ± SD, respectively. The Mann-Whitney test was used to compare EAE clinical scores. Student’s t test was used to compare continuous data. Correlation analysis involved the use of linear regression and Pearson correlation coefficients. The above analyses were carried out with GraphPad Prism 8.

## Results

### Generation of Smek1 knockout mice

Smek1 knockout mice were generated using the loxP-*Cre* system (Fig. S1a). The essential coding sequences in exon 2 of *Smek1* that were flanked by loxP sites were removed by *Cre*-mediated recombination. The detailed mating strategies are described in Fig. S1b. In brief, Smek1^-/+^ mice were obtained by mating Smek1^fl/fl^ mice with *Sox2-Cre* mice. *Sox2* is expressed in epiblasts, primordial germ cells, and postnatal oocytes [31, 32], and thus, the loxP-*Sox2-Cre* system could constitutively remove Smek1 from Smek1^-/+^ reciprocal cross offspring. The genotyping results of mouse tails are shown in Fig. S1c. Substantial prenatal death of Smek1^-/-^ mice was observed in the C57BL/6 strain (data not shown). Therefore, C57BL/6 Smek1^-/+^ mice were used for further animal experiments. The knockout efficiencies were examined by western blot analysis (Fig. S1d).

### SMEK1 reduction causes worse symptoms in the EAE model

**Fig. 1:**
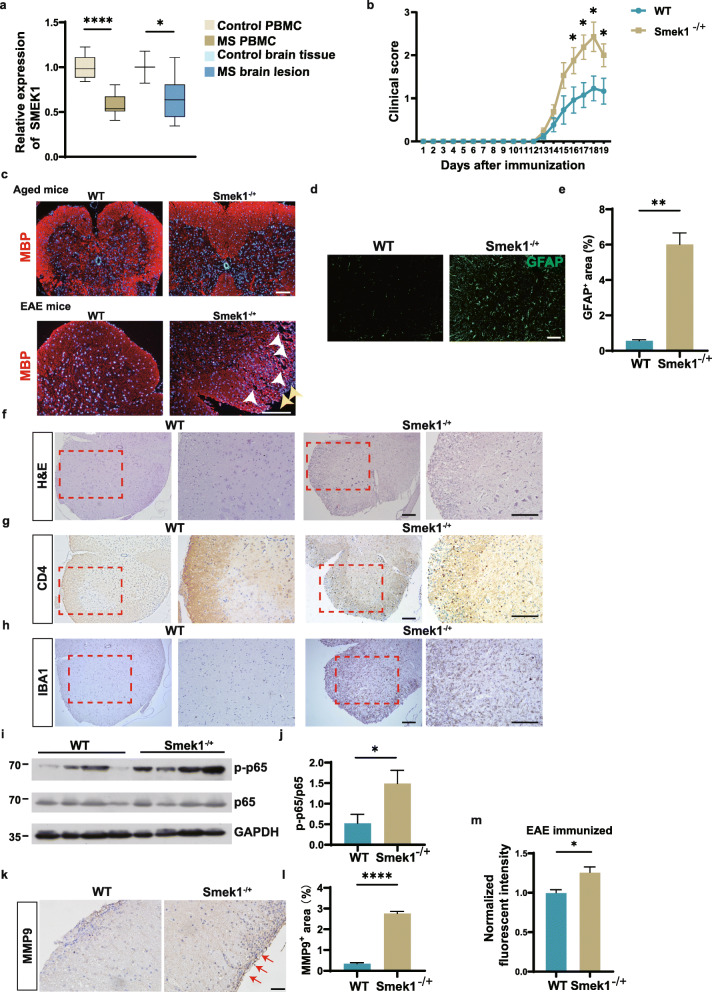
Suppressor of MEK1 (Smek1) reduction is related to multiple sclerosis (MS) and causes worsened experimental autoimmune encephalomyelitis (EAE) symptoms. **a** Box plot showing the transcriptional analysis of peripheral blood mononuclear cells from controls (n = 15), MS patients (n = 14), normal brain tissue (n = 3), and MS lesions (n = 9) from the Gene Expression Omnibus (GEO) dataset. PBMC, peripheral blood mononuclear cells. The box and whiskers are the median, minimum and maximum data for each group. Data are presented as the mean ± SEM and were analyzed by the two-sided unpaired t test; *P < 0.05; ****P < 0.0001. **b** Wild-type (n = 14) and Smek1^-/+^ (n = 17) mice were immunized with myelin oligodendrocyte glycoprotein 35-55 (MOG_35-55_) and CFA in 3 independent experiments. Daily clinical scores are shown as the mean ± SEM and were analyzed by the Mann-Whitney test, *P < 0.05. **c** Myelin basic protein (MBP) immunofluorescent staining of spinal cords from aged (10-month-old) mice and EAE mice. The white arrowheads indicate ruptured myelin; the yellow arrowheads indicate infiltrated cells (scale bar, 100 μm). **d** Immunofluorescent staining of spinal cords showing activated astrocytes (scale bar, 50 μm). **e** Quantification of GFAP staining in EAE spinal cords (n = 5 in each group). Data are presented as the mean ± SEM and were analyzed by the two-sided unpaired t test; **P < 0.01. **f**–**h** Hematoxylin-eosin (H&E) staining (**e**) and CD4 (**f**) and IBA1 (**g**) staining of spinal cord serial sections dissected from EAE spinal cords (scale bar, 100 μm). **i** Activation of the nuclear factor kappa B (NF-κB) signal detected by immunoblotting EAE brain tissue. **j** Western blot analysis was performed to evaluate the level of p-p65 (n = 8 in each group). Data are presented as the mean ± SEM and were analyzed by the two-sided unpaired t test; *P < 0.05. **k** Immunohistochemical staining of spinal cords for matrix metalloproteinase 9 (MMP9) levels. The red arrows point to dark stained tissue (scale bar, 50 μm). **l** Quantification of MMP9 deposition area in WT and Smek1^-/+^ EAE spinal cords (n = 5 in each group). Data are presented as the mean ± SEM and were analyzed by the two-sided unpaired t test; ****P < 0.0001. **m** Sodium fluorescein assay quantifying blood-brain barrier permeability in EAE mice (right) (n = 3 in each group) Data are presented as the mean ± SEM and were analyzed by the two-sided unpaired t test; *P < 0.05

To investigate the role of SMEK1 in CNS autoimmune disorders, we first used GEO transcriptional data from peripheral blood and brain tissue from normal controls and MS patients. The SMEK1 levels were reduced in both human MS peripheral blood mononuclear cells and MS brain lesions (Fig. 1a). To further investigate the role of Smek1 in autoimmune demyelination, we established an EAE model by MOG_35-55_ immunization of both Smek1^-/+^ mice and wild-type littermates. Symptoms occurred in both groups at day 13 postimmunization (Fig. 1b). Throughout the whole disease course, the clinical scores were higher for Smek1^-/+^ mice than for control mice. The clinical scores of mice in both groups peaked on day 18 postimmunization, and the mean clinical scores were 2.429 ± 0.091 and 1.231 ± 0.079 for Smek1^-/+^ and control mice, respectively. We first eliminated myelin degeneration caused by aging due to partial loss-of function of Smek1. No apparent demyelination was observed in aged mice (Fig. 1c, upper panel). Myelin basic protein (MBP) staining of EAE spinal cords revealed numerous demyelinated foci and infiltrated cell clusters in Smek1^-/+^ EAE mice (Fig. 1c, lower panel). Furthermore, astrocytes were more activated in EAE Smek1^-/+^ mice than in control mice (Fig. 1d, e). H&E staining revealed a greater number of infiltrated cells in the spinal cords of Smek1^-/+^ mice than in the spinal cords of control mice (Fig. 1f, Fig. S2a), which agreed with the degree of paralysis during the disease course. To specify the types of infiltrated cells in the spinal cord, we performed immunohistochemical staining of serial slices. Compared to wild-type mice, Smek1^-/+^ mice showed a slightly increased number of CD4-positive cells among the massive infiltrating cells (Fig. 1g, Fig. S2b). Nevertheless, most of the infiltrated cells in Smek1^-/+^ spines were CD4-negative. IBA1-positive cells, which represented both resident microglia and macrophages, were markedly accumulated in Smek1^-/+^ mice (Fig. 1h, Fig. S2c). On the other hand, the increase in P65 phosphorylation in Smek1^-/+^ cells indicated activation of the NF-κB pathway (Fig. 1i, j) and thus induced downstream collagenase matrix metalloproteinase (MMP9) expression (Fig. 1k, l). We next carried out blood-brain barrier assay in EAE mice. Compared to wild-type mice, Smek1^-/+^ mice showed higher fluorescence in brain homogenates 30 min after intraperitoneal injection (Fig. 1 m). These data indicate that the heterozygosity of Smek1 in EAE mice resulted in more inflammatory infiltration, severe pathological damage and worse clinical manifestations.

### Smek1 heterozygosity promotes microglial activation and CNS inflammation

**Fig. 2:**
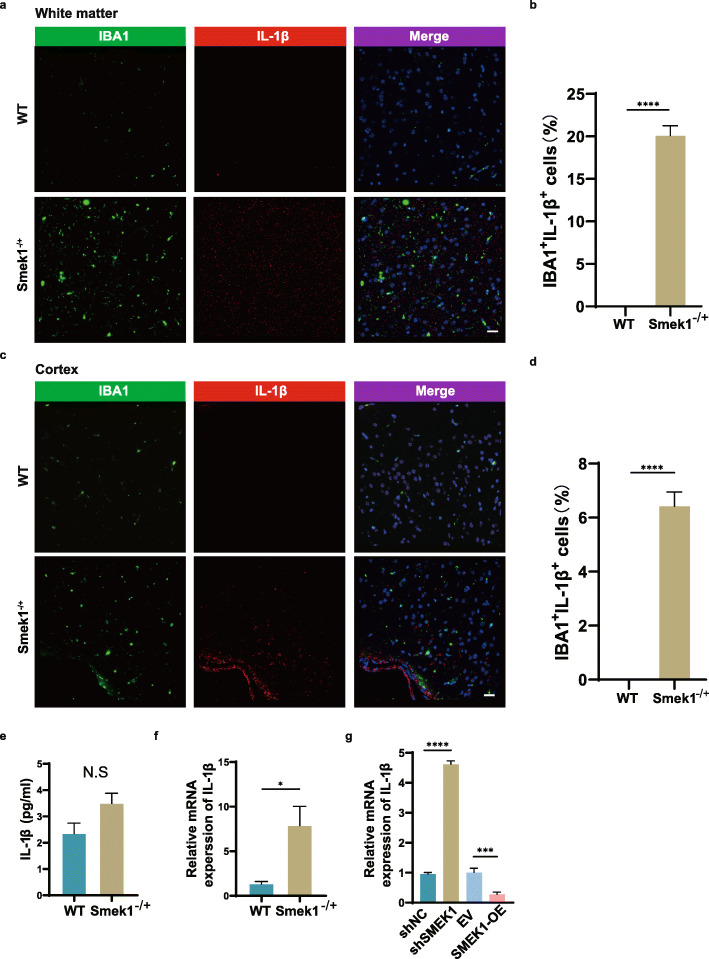
Microglial activation in EAE Smek1 heterozygotes. **a**, **b** IBA1 and IL-1β immunofluorescent staining (**a**) and quantification of IBA1^+^ IL-1β^+^ cells (**b**) of white matter from EAE mice sacrificed at 18 days postimmunization (scale bar, 50 μm). Data are presented as the mean ± SEM from 5 samples in each group and were analyzed by the two-sided unpaired t test. **c**, **d** IBA1 and IL-1β immunofluorescent staining (**c**) and quantification of IBA1^+^ IL-1β^+^ cells (**d**) of cortex tissue from EAE mice sacrificed at 18 days postimmunization (scale bar, 50 μm). Data are presented as the mean ± SEM from 5 samples in each group and were analyzed by the two-sided unpaired t test. **e** ELISA of the total IL-1β level in total brain tissue from EAE mice sacrificed at 18 days postimmunization (n = 4 in wild-type, n = 6 in Smek1^-/+^ mice). Data are presented as the mean ± SEM and were analyzed by the two-sided unpaired t test. **f** qPCR of the mRNA expression of IL-1β in EAE total brain tissue (n = 7 in both groups). Data are presented as the mean ± SEM and were analyzed by the two-sided unpaired t test; *P < 0.05. **g** qPCR of the mRNA level of IL-1β detected in an LPS-treated human microglial cell line (HMO6). Data are presented as the mean ± SD and were analyzed by the two-sided unpaired t test; ***P < 0.001; ****P < 0.0001

Since heterozygous Smek1 showed an accumulation of IBA1-positive cells in EAE tissue samples, we hypothesized that the heterozygosity of Smek1 expression could be sufficient to promote the activation of microglia to mediate neuroinflammation. Compared with wild-type mice, costaining of IBA1 and IL-1β showed a greater number and higher intensity of activated microglia in the white matter (Fig. 2a, b), cortex (Fig. 2c, d), and spinal cord (Fig. S3a, b) of EAE Smek1^-/+^ mice. ELISA of EAE brain tissue showed a slight elevation of the IL-1β level, however with no significance, in Smek1^-/+^ mice compared with wild-type mice (Fig. 2e). Additionally, mRNA expression of brain IL-1β was elevated in EAE Smek1^-/+^ mice compared with that in wild-type mice (Fig. 2f). Next, we established plvx-SMEK1-puro and shSMEK1 cell lines in the human microglial cell line HMO6. The knockdown and overexpression efficiencies were confirmed through western blotting (Fig. S3c, d). The HMO6 shSMEK1-2 cell line (hereafter referred to as shSMEK1) was used in further studies due to its higher knockout efficiency than the HMO6 shSMEK1-1 cell line. The IL-1β mRNA levels in SMEK1-OE, shSMEK1 and their corresponding controls were analyzed by qPCR. In line with previous results, IL-1β mRNA was elevated in shSMEK1 HMO6 cells and decreased in SMEK1-overexpressing cells (Fig. 2 g).

### Smek1 deficiency leads to the formation of a distinct proinflammatory microglial cluster

**Fig. 3:**
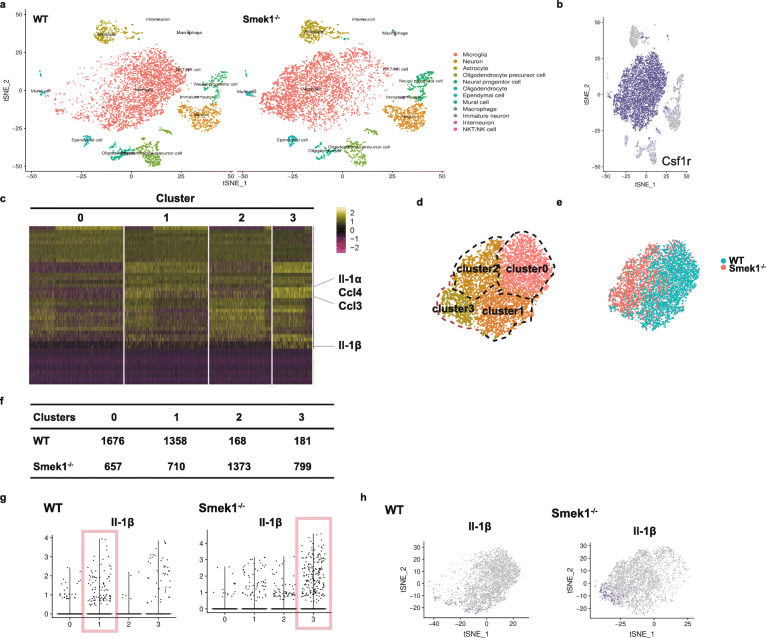
Single-cell RNA-seq of the brain cortex and hippocampus reveals hyperactivated microglia in Smek1^-/-^ mice. **a** tSNE visualization of individual cell clusters from the cortex and hippocampus of wild-type (n = 2) (left panel) and Smek1^-/-^ (n = 2) (right panel) mice. **b** tSNE plots highlighting the marker gene Csf1r for microglia. **c** Heatmap of total microglial subclusters. **d** Microglia are divided into 4 clusters according to diverse transcriptional patterns, namely, clusters 0 to 3. **e** Contribution of wild-type versus Smek1^-/-^ mouse samples to microglial cell clusters. **f** Cell number of microglial clusters in different genotypes. **g** Violin plots representing the distribution of the log-transformed normalized gene expression of IL-1β in microglia (clusters 0–3). The red box represents proinflammatory microglia. **h** tSNE map representing the distribution of the log-transformed normalized gene expression of IL-1β in microglia (clusters 0–3)

Single-cell transcriptome analysis was carried out in cortical and hippocampal samples from wild-type and Smek1^-/-^ mice (n = 2 in each group, 2 months old). Analysis of the ∼12,000 single-cell transcriptomes revealed 12 cell subtypes (Fig. 3a). Microglia were distinguished by a microglia-specific marker, colony-stimulating factor 1 receptor (Csf1r) (Fig. 3b). The expression heat map of total microglia suggested that although total microglia in wild-type and Smek1^-/-^ mice showed similar expression patterns, an internal transcriptional diversity was observed that could be used to divide them into several clusters (Fig. 3c). For instance, the proinflammatory cytokines interleukin 1 alpha (IL-1α), IL-1β, Ccl3, and Ccl4 were enriched in cluster 3. Based on the gene expression of variable features, clustering of mouse total microglia identified four populations in the tSNE map (Fig. 3d). When labeled by origin, microglia in wild-type and Smek1^-/-^ mice demonstrated a distinct topographical distribution (Fig. 3e). Most wild-type microglia tended to be classified into clusters 0 and 1, whereas Smek1^-/-^ microglia had fewer cells in clusters 0 and 1 but more cells in clusters 2 and 3 (Fig. 3f). In addition, we observed a higher expression level of IL-1β (Fig. 3 g) and more IL-1β-positive cells (Fig. 3 h) in Smek1^-/-^ cluster 3 than in wild-type clusters. The above evidence indicated the presence of a preactivated and proinflammatory microglial cluster in the Smek1^-/-^ CNS.

**Fig. 4:**
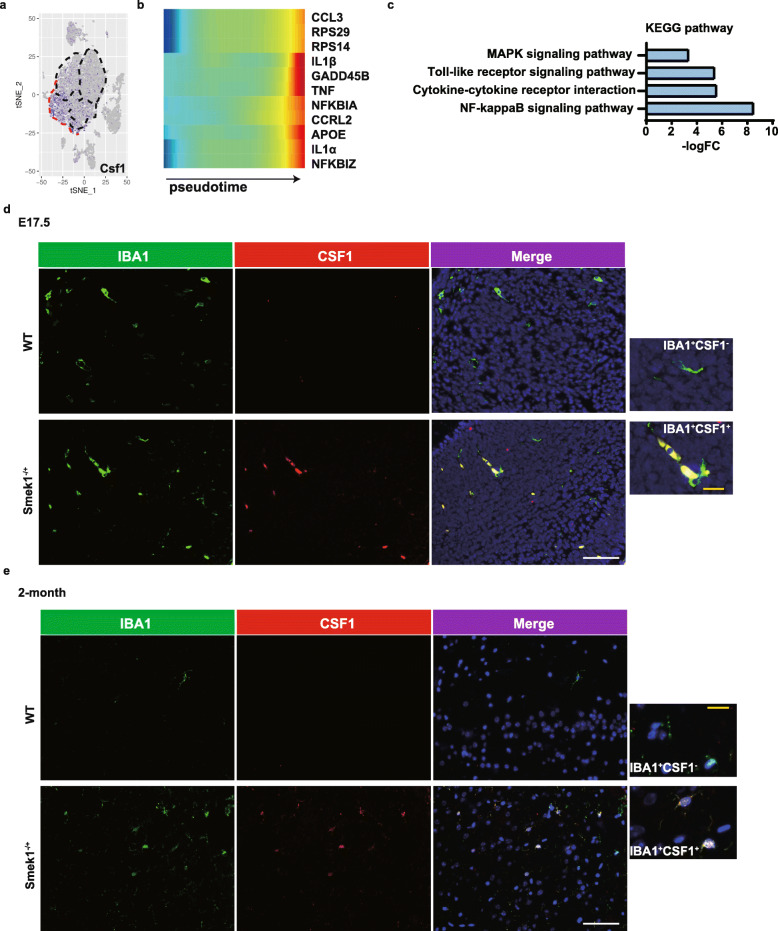
Proinflammatory colony stimulating factor 1 (Csf1)-positive microglia in the Smek1-deficient central nervous system (CNS). **a** tSNE plots of Csf1 expression in total microglia. **b** Heatmap of pseudotime gene expression in Smek1^-/-^ microglia. **c** Kyoto Encyclopedia of Genes and Genomes (KEGG) analysis of marker genes in cluster 3. **d**, **e** Colony stimulating factor 1 (Csf1) staining in wild-type and Smek1^-/+^ mice at E17.5 (**d**) and 2 months old (**e**) (white scale bar, 25 μm). IBA1^+^Csf1^-^ microglia in wild-type (upper) and IBA1^+^Csf1^+^ microglia in the Smek1^-/+^ cortex (lower) are magnified (yellow scale bar, 5 μm)

Subpopulation markers were identified to reveal the heterogeneity of cluster 3. Further analysis revealed enrichment of macrophage Csf1 in cluster 3 (Fig. 4a). Csf1 is a key regulator of myeloid lineage cells and supports distinct subpopulations of microglia [33]. A heatmap of the pseudotime analysis results of Smek1-deficient microglia indicated upregulation of proinflammatory cytokines, including IL-1β (Fig. 4b). KEGG enrichment of marker genes in cluster 3 displayed genes related to inflammation pathways, including the mitogen-activated protein kinase (MAPK) and NF-κB pathways (Fig. 4c).

However, as mentioned above, substantial prenatal death was found for Smek1^-/-^ fetuses. Based on the evidence from Smek1^-/-^ mice, we investigated the spatial and temporal expression features of Csf1 in Smek1^-/+^ microglia. Csf1 was stained in brain tissue samples from embryonic day (E17.5) (Fig. 4d) and 2-month-old Smek1^-/+^ and wild-type littermates (Fig. 4e). In Smek1^-/+^ mice, we observed both Csf1-positive and Csf1-negative microglia (IBA1-positive) in E17.5 and adult brains. These findings confirmed that with partial or complete loss of function of Smek1, a novel Csf1^+^ microglial cluster had a preactivated phenotype that may promote neuroinflammation.

### Smek1 heterozygosity promotes macrophage activation

**Fig. 5:**
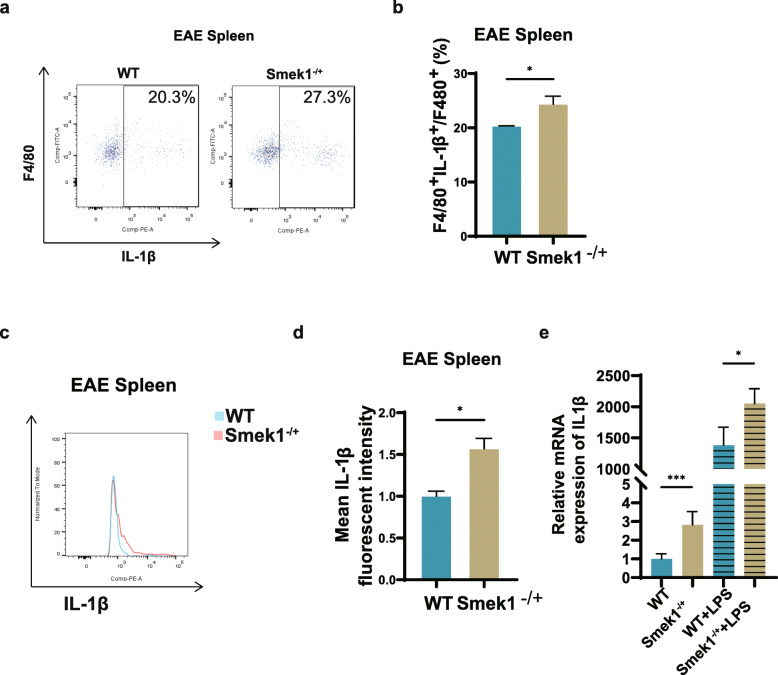
Smek1 heterozygosity upregulates macrophage IL-1β expression in both the resting and activated states. **a**, **b** Flow cytometry of IL-1β^+^ macrophages with anti-mouse F4/80 and IL-1β antibodies (n = 3 control, n = 4 Smek1^-/+^) (**a**); data are shown in the bar graph (**b**). **c**, **d** Mean fluorescence intensity of IL-1β in splenic IL-1β + macrophages (n = 3 control, n = 4 Smek1-/+) (c); data are shown in the bar graph (**d**). **e** qPCR analysis of the relative expression of macrophage cytokines in cultured mononuclear cells with and without lipopolysaccharide (LPS) treatment. Data are presented as the mean ± SEM and were analyzed by the two-sided unpaired t test; *P < 0.05; ***P < 0.001

Since microglia derives from yolk sac macrophages, which could also differentiate into peripheral macrophages, we hypothesized that Smek1^-/+^ macrophages also contributed to neuroinflammation. First, the proportion of F4/80^+^ IL-1β^+^ macrophages in the periphery (Fig. 5a, b) and the macrophage IL-1β mean fluorescence intensity (Fig. 5c, d) in the spleen were elevated in Smek1 heterozygotes. To further investigate the characteristics of Smek1^-/+^ myeloid cells, spleens from 6- to 8-week-old mice were dissected and ground. Cells were cultivated in 1640 medium with and without LPS for 48 h. Adherent cells were collected for qPCR. The results showed an elevated IL-1β level in the LPS-treated group compared to the wild-type group (Fig. 5e). Interestingly, the level of IL-1β was also increased in the naïve group. Consistent with the features observed in Smek1-deficient microglia, Smek1^-/+^ macrophages were also preactivated in the “resting” state and contributed to inflammation onset by overexpressing IL-1β.

### Smek1 is required for IDO1 maintenance by regulating the IFN-γ/STAT1 axis

**Fig. 6:**
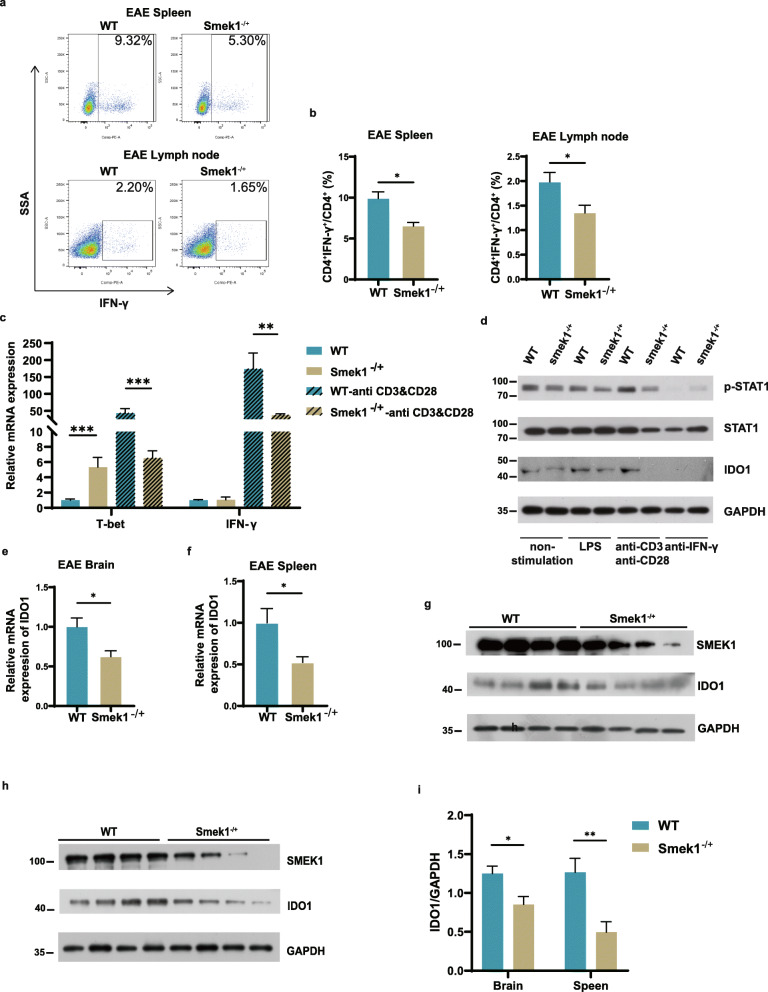
Smek1 is required for indoleamine 2,3-dioxygenase 1 (IDO1) maintenance in the immune system and CNS. **a**, **b** IFN-γ^+^ splenic and lymph node CD4^+^ Th1 cells were detected by flow cytometry (n = 7 in both groups) (left panel); data are shown in the bar graph (right panel). **c** qPCR analysis of the relative expression of T-bet and IFN-γ with and without anti-CD3&CD28 treatment. **d** Western blot and quantification analyses of the protein levels in splenic mononuclear cells with no stimulation and treatment with LPS and the anti-CD3&CD28 and anti-IFN-γ antibodies. **e** qPCR analysis of the mRNA level of IDO1 in the EAE brain (n = 9 in control, n = 8 in Smek1^-/+^ mice). **f** qPCR analysis of the mRNA level of IDO1 in the EAE spleen (n = 9 in control, n = 8 in Smek1^-/+^ mice). **g** Western blot and quantification analyses of the IDO1 protein levels in the EAE brain. **h** Western blot and quantification analyses of IDO1 protein levels in the EAE spleen. **i** Representative western blot analysis of IDO1 expression level in EAE brain and spleen (n = 8 in each group). Data are presented as the mean ± SEM and were analyzed by the two-sided unpaired t test; *P < 0.05; **P < 0.01; ***P < 0.001

Since the activation of CD4 Th cells specific for antigens in the myelin sheath is essential to the pathogenesis of MS, we next examined whether CD4-positive T cells participated in exacerbating EAE in Smek1^-/+^ mice. Compared with control mice, the proportions of CD4^+^ IFN-γ^+^ cells among CD4^+^ cells in the spleen and lymph nodes were greatly reduced in Smek1^-/+^ mice (Fig. 6a, b). This finding is contradictory to the conventional concept of Th1-mediated EAE. An in vitro study of splenic mononuclear cells revealed that upon anti-CD3&CD28 stimulation, T-bet and IFN-γ were significantly reduced in Smek1^-/+^ cells (Fig. 6c). Western blot analysis showed that IDO1 and its transcription factor p-STAT1 were decreased in Smek1^-/+^ cells when treated with anti-CD3&CD28 or LPS (Fig. 6d, Fig. S4a, b). Additionally, IFN-γ/STAT1-dependent IDO1 activity was confirmed by an anti-IFN-γ antibody treatment. Blocking IFN-γ with a specific antibody resulted in IDO1 ablation in both groups.

Next, we examined the IDO1 mRNA and protein levels in EAE spleen and brain tissues. Consistent with the low IFN-γ in Th1 cells, IDO1 mRNA and protein level was downregulated in both the brain (Fig. 6e, g) and spleen (Fig. 6f, h) of Smek1^-/+^ mice (Fig. 6i). Taken together, our results suggest that when Smek1 is partially depleted, Th1 cells downregulate IFN-γ when activated, resulting in the suppression of IDO1 expression through the IFN-γ/STAT1 axis.

### Inactivation of IDO1-AhR signaling impairs immunosuppression in Smek1-/+ myeloid cells

**Fig. 7:**
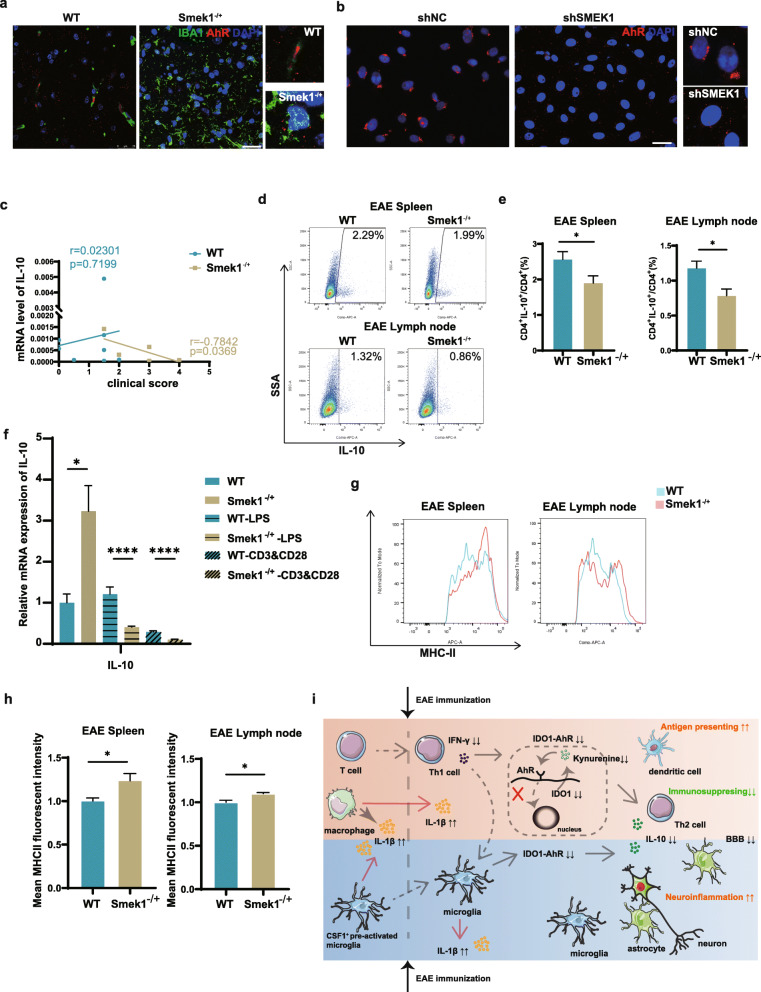
Inactivation of the IDO1-aryl hydrocarbon receptor (AhR) pathway promotes neuroinflammation in Smek1 heterozygous mice. **a** AhR subcellular localization in spinal microglia detected by IBA1 and AhR costaining (scale bar, 25 μm). **b** Immunofluorescent staining of AhR in HMO6 cells treated with LPS (scale bar, 25 μm). **c** Correlation between IL-10 expression in the EAE brain and the clinical scores (r and p values are presented for wild-type and Smek1^-/+^ mice data in the corresponding colors). The linearity of the correlation was tested with Pearson’s correlation. **d**, **e** Flow cytometry of the IL-10 level (**d**) and its quantification (**e**) in Th2 cells. Th2 cells were labeled with CD4 and IL-4. Data are presented as the mean ± SEM and were analyzed by the two-sided unpaired t test; *P < 0.05. **f** qPCR analysis of IL-10 in cultured mononuclear cells with and without treatment with LPS and anti-CD3&CD28. Data are presented as the mean ± SEM and were analyzed by the two-sided unpaired t test; *P < 0.05; ****P < 0.0001. **g**, **h** Mean MHC-II fluorescence intensity in the spleen (g) (n = 8 control, n = 6 Smek1^-/+^) and lymph nodes (h) (n = 8 control, n = 6 Smek1^-/+^). DCs were detected by labeling of CD11c and MHC-II. Data are presented as the mean ± SEM and were analyzed by the two-sided unpaired t test; *P < 0.05. **i** Schematic model of EAE pathogenesis in Smek1 heterozygotes

We hypothesized that downregulation of the IDO1-AhR pathway might further interfere with the maintenance of immunosuppression in Smek1^-/+^ mice and elicit pronounced inflammation during EAE. To verify this hypothesis, we first detected the subcellular location of AhR microglia. Reduced IDO1 activity may cause dysfunction in AhR nuclear translocation. The results showed that the AhR signal was reduced in microglial nuclei in the spinal cords of EAE Smek1^-/+^ mice (Fig. 7a). This finding was further confirmed in LPS-treated HMO6 cells. LPS stimulation caused a nuclear or near-nuclear distribution of AhR in control microglia but dispersed AhR molecules in HMO6 cells transfected with SMEK1 shRNA (Fig. 7b). AhR is a known positive transcription factor of IL-10 that may regulate immune responses in EAE. Thus, we examined IL-10 mRNA expression in EAE brains and correlated it with the clinical scores of each corresponding mouse. In Smek1^-/+^ mice, the clinical score was inversely associated with the IL-10 level; however, this negative relationship was not observed in wild-type mice (Fig. 7c). Moreover, reduced IL-10 levels were detected in splenic and lymph node Th2 cells from Smek1^-/+^ mice (Fig. 7d, e). This result was further confirmed by an in vitro study. As shown in Fig. 7f, IL-10 was elevated in Smek1^-/+^ mice in steady state. When stimulated with LPS or anti-CD3&CD28, IL-10 was reduced in the Smek1^-/+^ group.

We next examined the tolerogenic role of IFN-γ resulted in DCs. In Smek1^-/+^ EAE mice, the mean fluorescence intensity of MHC-II was increased in splenic and lymph node DCs compared to that in splenic and lymph node DCs in wild-type mice (Fig. 7g, h).

In conclusion, the inhibition of the IDO1-AhR immunosuppressive cascade in myeloid cells, caused by low IFN-γ secretion upon Th1 activation, is closely related to worsened clinical symptoms in Smek1^-/+^ EAE mice.

## Discussion

Here, we focused on the role of Smek1 in neuroinflammation. After discovering that SMEK1 is expressed at low levels in MS patients, we speculated that Smek1 reduction might contribute to susceptibility to CNS autoimmune diseases. To verify our hypothesis, we established an EAE model in Smek1^-/+^ mice and their littermates. Smek1^-/+^ mice showed worsened symptoms than their littermates, and IL-1β was elevated in myeloid cells. By single-cell RNA sequencing, we identified a preactivated Csf1^+^ microglial subpopulation in Smek1-deficient mice, possibly resulting from abnormal embryonic myeloid development, which may promote CNS inflammation. We also revealed that the IDO1-AhR pathway was inhibited due to the downregulation of IFN-γ in Smek1^-/+^ Th1 cells (Fig. 7i).

In multiple sclerosis, microglia are responsible for antigen presentation and accumulate in demyelinating lesions [11, 14]. A study of MS-associated genes revealed distinct expression patterns in microglia from gray and white matter [34]. Compared with white matter microglia, gray matter microglia in the MS group showed higher expression of genes related to inflammation. Our single-cell transcriptional analysis of the brain cortices and the hippocampus revealed a unique Csf1^+^ microglial cluster that highly expresses IL-1β. Embryonic and postnatal development of macrophages and microglia largely depends on CSF1-CSF1R signaling [35] CSF1 regulates macrophage differentiation and is the most potent factor controlling the development, survival, and local self-renewal of the microglial population [36]. Previous studies have revealed that CSF1 is upregulated in an EAE model, and blocking the CSF1-CSF1R interaction shows therapeutic effects [37, 38]. Furthermore, CSF1 overexpression induces microglial activation and IL-1β secretion [39]. Thus, we speculated that high expression of Csf1 resulted in proinflammatory microglia in Smek1 heterozygotes, which subsequently promoted CNS inflammation after establishing EAE. Interestingly, heterozygous Smek1 macrophages also showed high IL-1β before and after EAE immunization. In fact, microglia and macrophages, both of which are mononuclear phagocytes, originate from precursors with erythromyeloid potential, which give rise to different myeloid cell types. This finding suggests that partial loss of Smek1 promotes myeloid cell activation and mediates the onset and development of autoimmune demyelination. Thus, Smek1 is likely to be a risk factor of MS and may be instructive in regard to early intervention.

In the immune system, stimulation with exogenous or T-cell–derived IFN-γ promotes DCs to generate functional IDO [40]. In terms of maintaining immune tolerance, IDO1 plays a pivotal role in the tumor microenvironment and autoimmune diseases [41]. In an EAE model, Xiao et al. first reported the therapeutic potential of IFN-γ–modified DCs with elevated IDO1 mRNA levels [27]. IDO1-mediated degradation of tryptophan caused accumulation of its metabolite, kynurenine, which binds to and activates AhR [42]. Here, in the EAE model, we observed lower IFN-γ expression in Smek1^-/+^ T cells, accompanied by dysfunction of immunosuppression of the IDO1-AhR pathway. In vitro study showed that when T cells were specifically activated, IDO1 was dramatically suppressed in Smek1^-/+^ group (Fig. 7d). We suppose that the reduced IFN-γ expression in Smek1^-/+^ T cells upon activation resulted in low IDO1 and thus impaired the immunosuppression pathway.

Studies have revealed a transcriptional regulatory role of AhR in immune-related cells. AhR directly binds to and regulates the expression of IL-10 in IL-10–producing regulatory B cells and IL-10–producing regulatory type 1 T cells [43, 44]. In our study, Smek1^-/+^ Th2 cells had a lower level of the anti-inflammatory cytokine IL-10. We also detected IL-10 expression level using EAE tissue. Interestingly, IL-10 mRNA level is lower in Smek1^-/+^ group and is negatively correlated with clinical score. Given that the kynurenine pathway also exists in microglia [45–47], we speculated that kynurenine-dependent activation of AhR could also affect IL-10 expression in Smek1^-/+^ microglia.

The results of our study displayed a detrimental role of low IFN-γ in autoimmune demyelination. In fact, the preconception of the proinflammatory role of IFN-γ has been challenged by its paradoxical characteristics in certain autoimmune diseases. Research using experimental models and patients has revealed the immunoregulatory function of IFN-γ in specific autoimmune diseases during specific stages [48, 49]. In Lewis rats, local administration of IFN-γ to the CNS suppressed clinical signs, whereas anti-IFN-γ antibody treatment before disease onset caused more severe symptoms [19]. Injecting anti-IFN-γ antibodies into EAE C57BL/6J mice increased disease morbidity and mortality, while IFN-γ treatment in mice ameliorated disease severity [16]. In MS patients, anti-IFN-γ therapy in secondary progressive MS showed optimistic results [18]. Yet, we lack an optimum dose or time of administration that could have clinical implications [48] In our study, we believe that IFN-γ is downregulated in Smek1 heterozygotes, as inhibition of its tolerogenic function contributes to an aggravated EAE phenotype. This result is likely applicable to MS, where low IFN-γ may be responsible for the neuroinflammation in patients with low Smek1 level. Thus, Smek1 expression level may be an instructive factor when selecting IFN-γ treatment in MS patients. However, the underlying mechanism of impaired IFN-γ expression in Smek1^-/+^ Th1 cells upon activation is still unclear. The dynamic changes of IFN-γ level in Smek1^-/+^ along with the course of the disease is also essential for unveiling the function of IFN-γ in neuroinflammation. Thus, IFN-γ for treating MS is far more complicated and needs to be studied according to dose- and stage-specific circumstances.

## Conclusions

In summary, SMEK1 plays a pivotal role in maintaining microglial homeostasis by mediating microglial preactivation. Meanwhile, SMEK1 is downregulated in both PBMCs and brain tissue from MS patients. Thus, Smek1 reduction, similar to Smek1 heterozygosity, might provide a sensitive background for developing CNS autoimmune disease in the setting of other genetic or environmental factors. Smek1 might represent as a possible target for developing early interventional treatments of MS.

## Supplementary Information

**Additional file 1.** Fig. S1a. Smek1 knockout mice generated using the loxP-Cre system. Fig. S1b. Detailed mating strategies. Fig. S1c. Genotyping results of mouse tails. Fig. S1d. Knockout efficiencies examined by western blot analysis.

**Additional file 2.** Fig. S2a. H&E staining revealing a greater number of infiltrated cells in the spinal cords of Smek1-/+ mice than in the spinal cords of control mice. Fig. S2b. Smek1-/+ mice showed a slightly increased number of CD4-positive cells among the massive infiltrating cells. Fig. S2c. IBA1-positive cells were markedly accumulated in Smek1-/+ mice.

**Additional file 3.** Fig. S3. Immunostaining of EAE spinal cords microglia and establishing HMO6 transfected cell line.(a) Immunofluorescent staining of EAE spinal cords showing activated IL-1β–positive microglia in Smek1-/+(Scale bar, 50 μm). Yellow arrow heads pointed to IBA1-positive microglia with no IL-1β signals. Whitearrow heads pointed to IBA1+IL-1β+ microglia. All cells indicated by arrow heads are magnified anddisplayed on the right.(b) Quantification of IBA1+IL1β+ staining of spinal cords obtained from EAE mice.(n=6 in each group)(c) Western blot of SMEK1 in HMO6 cell line transfected with 2 different shSMEK1 vectors and negative control.(d) Western blot of SMEK1 in HMO6 cell line transfected with SMEK1 overexpression vector and correspondingempty vector.Data are represented as mean ± SEM and were analyzed by the two-sided unpaired t test. ****, *p* < 0.0001.

**Additional file 4.** Fig.S4 Western blot analyses of protein levels in splenic mononuclear cells. (a) Western blot analysis of STAT1 phosphorylation level in splenic mononuclear cells (*n* = 8 in each group). (b) Western blot analysis of IDO1 protein level in in splenic mononuclear cells (*n *= 8 in each group). Data are represented as mean ± SEM and were analyzed by the two-sided unpaired t test. **, *p *< 0.01; ****, *p* < 0.0001.

## Data Availability

The data that supports the findings of this study is available from the corresponding author upon reasonable request.

## Abbreviations

AhR: Aryl hydrocarbon receptor
PP4: Protein phosphatase 4
AMPK: Adenosine monophosphate activated protein kinase
BBB: Blood–brain barrier
CNS: Central nervous system
Csf1: Colony-stimulating factor 1-positive
Csf1r: Colony-stimulating factor 1 receptor
MBP: Myelin basic protein
MHC: Major histocompatibility complex
MOG_35-55_: Myelin oligodendrocyte glycoprotein 35-55
EAE: Experimental autoimmune encephalomyelitis
GEO: Gene Expression Omnibus
HMO6: Human microglia cell line
IDO1: Indoleamine 2,3-dioxygenase 1
IFN-γ: Interferon γ
IL-1α: Interleukin 1 alpha
IL-1β: Interleukin 1β
MAPK: Mitogen activated protein kinase
MHC: Major histocompatibility complex
MS: Multiple sclerosis
NF-κB: Nuclear factor kappa B
SMEK1: Suppressor of MEK1
Th: T helper
