# Correction: Smek1 deficiency exacerbates experimental autoimmune encephalomyelitis by activating proinflammatory microglia and suppressing the IDO1-AhR pathway

**DOI:** 10.1186/s12974-023-02834-6

**Published:** 2023-07-06

**Authors:** Ruo-Nan Duan, Chun-Lin Yang, Tong Du, Ai Liu, An-Ran Wang, Wen-Jie Sun, Xi Li, Jiang-Xia Li, Chuan-Zhu Yan, Qi-Ji Liu

**Affiliations:** 1 Key Laboratory for Experimental Teratology of the Ministry of Education and Department of Medical Genetics, Cheeloo College of Medicine, School of Basic Medical Sciences, Shandong University, No. 44 West Wenhua Road, Jinan, Shandong 250012 People’s Republic of China; 2 Department of Neurology, Qilu Hospital, Cheeloo College of Medicine, Shandong University, Jinan, People’s Republic of China; 3 Department of Neurology, Shandong Provincial Qianfoshan Hospital, Cheeloo College of Medicine, Shandong University, Jinan, People’s Republic of China

**Correction** **:** **Journal of Neuroinflammation (2021) 18:145** **https://doi.org/10.1186/s12974-021-02193-0**

Following publication of the original article [1], the authors identified an error in “Method”. It has been corrected in this correction.

In “Materials and methods” section, under the heading “qPCR” part: 5′-CCTCTCTCTAATCAGCCCTCTG-3′ (reverse).

The reverse primer sequence of IL-1β (homo) should be 5′-GTCGGAGATTCGTAGCTGGA-3′.
