# Supplementary material for: Smek1 deficiency exacerbates experimental autoimmune encephalomyelitis by activating proinflammatory microglia and suppressing the IDO1-AhR pathway
Source: J Neuroinflammation. 2021 Jun 28;18:145. doi: 10.1186/s12974-021-02193-0 (PMC8237434; doi:10.1186/s12974-021-02193-0)
Supplement: Supplementary file 1 — Additional file 1. Fig. S1a. Smek1 knockout mice generated using the loxP-Cre system. Fig. S1b. Detailed mating strategies. Fig. S1c. Genotyping results of mouse tails. Fig. S1d. Knockout efficiencies examined by western blot analysis. [file 12974_2021_2193_MOESM1_ESM.pdf]

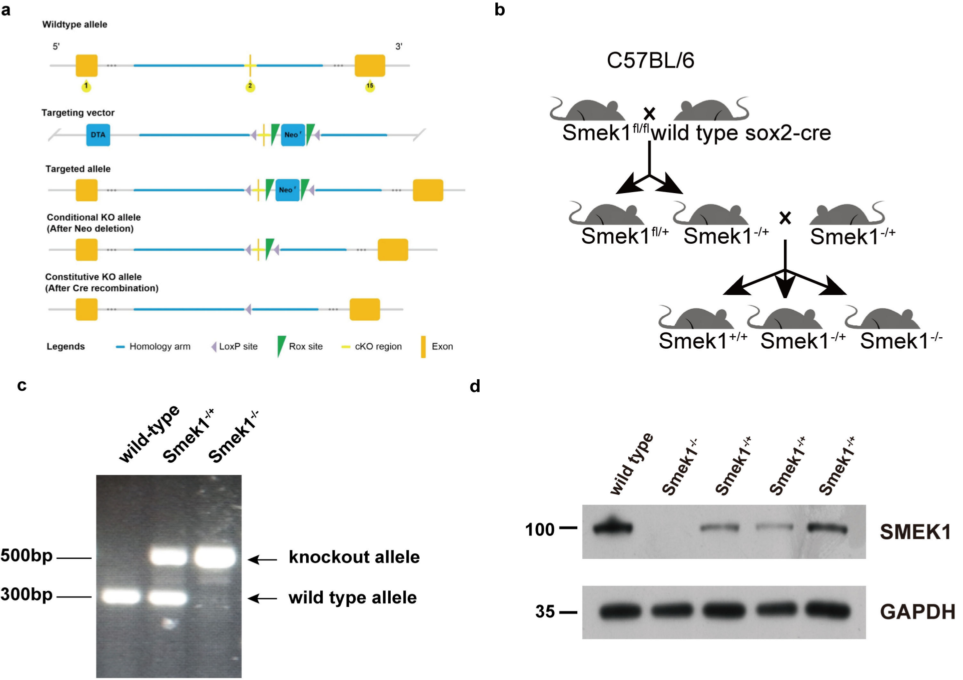

Fig.S1 Generation of Smek1 knockout mice.

(a) Generation of mice with loxP-flanked SMEK1 alleles through recombination in linearized SMEK1 vector.

(b) Mating strategies of Smek1 knockout mice.

(c) Genotype of Smek1$^{-/+}$ knockout mice.

(d) Western blot analysis of SMEK1 knockout efficiency in Smek1$^{-/+}$.
