# Supplementary material for: Smek1 deficiency exacerbates experimental autoimmune encephalomyelitis by activating proinflammatory microglia and suppressing the IDO1-AhR pathway
Source: J Neuroinflammation. 2021 Jun 28;18:145. doi: 10.1186/s12974-021-02193-0 (PMC8237434; doi:10.1186/s12974-021-02193-0)
Supplement: Supplementary file 2 — Additional file 2. Fig. S2a. H&E staining revealing a greater number of infiltrated cells in the spinal cords of Smek1-/+ mice than in the spinal cords of control mice. Fig. S2b. Smek1-/+ mice showed a slightly increased number of CD4-positive cells among the massive infiltrating cells. Fig. S2c. IBA1-positive cells were markedly accumulated in Smek1-/+ mice. [file 12974_2021_2193_MOESM2_ESM.pdf]

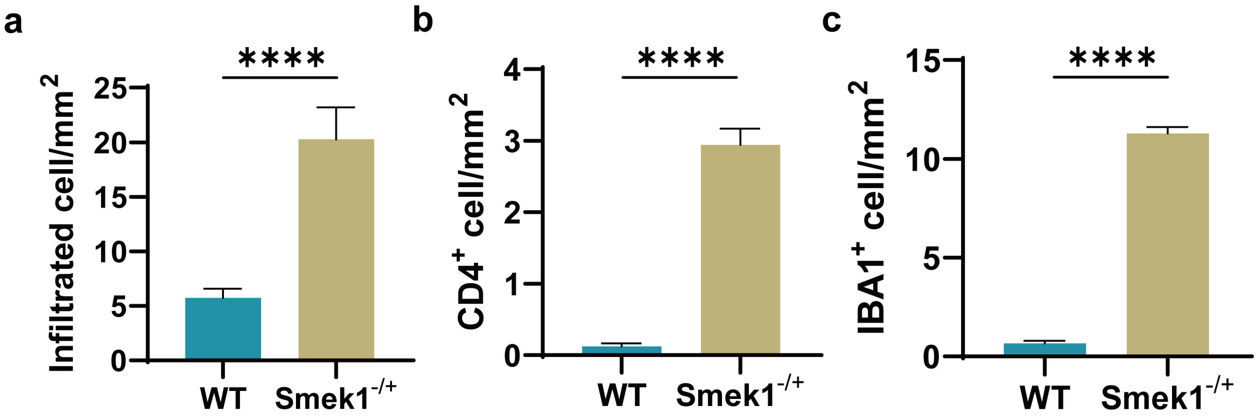

Fig.S2 Quantification of infiltrated cells in EAE spinal cords.
(a) Representative analysis of infiltrated cell counts in H&E stainings of EAE spinal cords (n=5 in each group).
(b) Representative analysis of CD4-positive cells in EAE spinal cords (n=5 in each group).
(c) Representative analysis of IBA1-positive cells in EAE spinal cords (n=5 in each group).

Data are represented as mean ± SEM and were analyzed by the two-sided unpaired t test. ****, $p < 0.0001$.
