# Supplementary material for: Smek1 deficiency exacerbates experimental autoimmune encephalomyelitis by activating proinflammatory microglia and suppressing the IDO1-AhR pathway
Source: J Neuroinflammation. 2021 Jun 28;18:145. doi: 10.1186/s12974-021-02193-0 (PMC8237434; doi:10.1186/s12974-021-02193-0)
Supplement: Supplementary file 3 — Additional file 3. Fig. S3. Immunostaining of EAE spinal cords microglia and establishing HMO6 transfected cell line.(a) Immunofluorescent staining of EAE spinal cords showing activated IL-1β–positive microglia in Smek1-/+(Scale bar, 50 μm). Yellow arrow heads pointed to IBA1-positive microglia with no IL-1β signals. Whitearrow heads pointed to IBA1+IL-1β+ microglia. All cells indicated by arrow heads are magnified anddisplayed on the right.(b) Quantification of IBA1+IL1β+ staining of spinal cords obtained from EAE mice.(n=6 in each group)(c) Western blot of SMEK1 in HMO6 cell line transfected with 2 different shSMEK1 vectors and negative control.(d) Western blot of SMEK1 in HMO6 cell line transfected with SMEK1 overexpression vector and correspondingempty vector.Data are represented as mean ± SEM and were analyzed by the two-sided unpaired t test. ****, p < 0.0001. [file 12974_2021_2193_MOESM3_ESM.pdf]

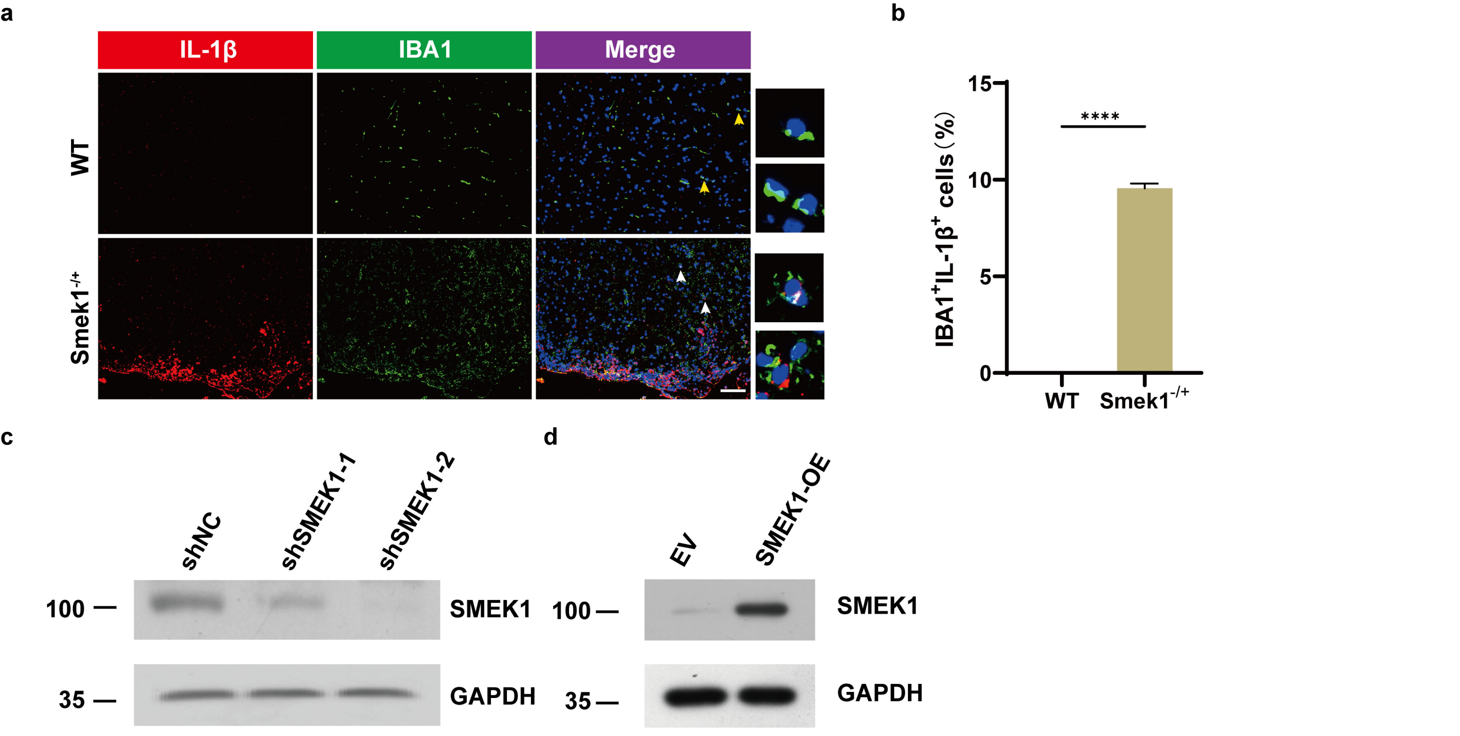

Fig.S3 Immunostaining of EAE spinal cords microglia and establishing HMO6 transfected cell line.

(a) Immunofluorescent staining of EAE spinal cords showing activated IL-1β–positive microglia in Smek1$^{-/+}$ (Scale bar, 50 μm). Yellow arrow heads pointed to IBA1-positive microglia with no IL-1β signals. White arrow heads pointed to IBA1+IL-1β+ microglia. All cells indicated by arrow heads are magnified and displayed on the right.

(b) Quantification of IBA1+IL1β+ staining of spinal cords obtained from EAE mice.(n=6 in each group)

(c) Western blot of SMEK1 in HMO6 cell line transfected with 2 different shSMEK1 vectors and negative control.

(d) Western blot of SMEK1 in HMO6 cell line transfected with SMEK1 overexpression vector and corresponding empty vector.

Data are represented as mean ± SEM and were analyzed by the two-sided unpaired t test. ****, $p < 0.0001$.
