# Supplementary material for: Smek1 deficiency exacerbates experimental autoimmune encephalomyelitis by activating proinflammatory microglia and suppressing the IDO1-AhR pathway
Source: J Neuroinflammation. 2021 Jun 28;18:145. doi: 10.1186/s12974-021-02193-0 (PMC8237434; doi:10.1186/s12974-021-02193-0)
Supplement: Supplementary file 4 — Additional file 4. Fig.S4 Western blot analyses of protein levels in splenic mononuclear cells. (a) Western blot analysis of STAT1 phosphorylation level in splenic mononuclear cells (n = 8 in each group). (b) Western blot analysis of IDO1 protein level in in splenic mononuclear cells (n = 8 in each group). Data are represented as mean ± SEM and were analyzed by the two-sided unpaired t test. **, p < 0.01; ****, p < 0.0001. [file 12974_2021_2193_MOESM4_ESM.pdf]

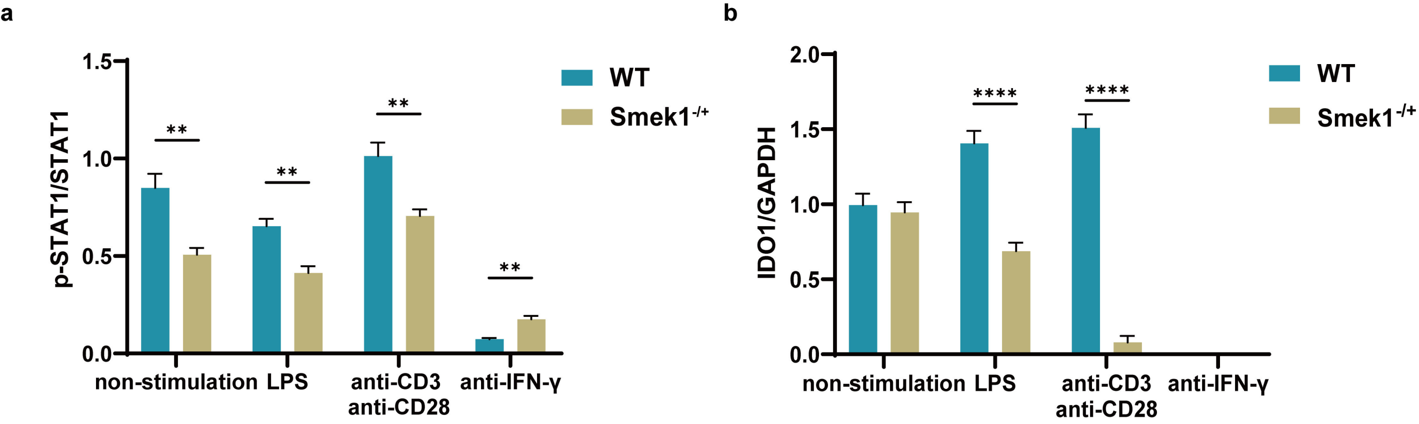

Fig.S4 Western blot analyses of protein levels in splenic mononuclear cells.
(a) Western blot analysis of STAT1 phosphorylation level in splenic mononuclear cells (n=8 in each group).
(b) Western blot analysis of IDO1 protein level in in splenic mononuclear cells (n=8 in each group).

Data are represented as mean ± SEM and were analyzed by the two-sided unpaired t test. **, p< 0.01; ****, p < 0.0001.
